# The Impact of Intrinsic Capacity on Quality of Life in Older Adults: A Multidimensional SEM Analysis

**DOI:** 10.1093/geroni/igaf122.2469

**Published:** 2025-12-31

**Authors:** Zhen Wu, Lulu Xiao, Junyan Zeng, Ershuo Zhao, Haijun Zhao, Shixing Song, Yan Dong, Linlin Hu

**Affiliations:** Chinese Academy of Medical Sciences & Peking Union Medical College, Beijing, China (People’s Republic); Chinese Academy of Medical Sciences & Peking Union Medical College, Beijing, China (People’s Republic); Chinese Academy of Medical Sciences & Peking Union Medical College, Beijing, China (People’s Republic); Donghai County People’s Hospital, Lianyungang, Jiangsu, China (People’s Republic); Ganyun County Traditional Chinese Medicine Hospital, Lianyungang, Jiangsu, China (People’s Republic); Guanyun County People’s Hospital, Lianyungang, Jiangsu, China (People’s Republic); Nanjing Medical University, Nanjing, Jiangsu, China (People’s Republic); Chinese Academy of Medical Sciences & Peking Union Medical College, Beijing, China (People’s Republic)

## Abstract

Amidst global population aging, while the WHO’s Intrinsic Capacity (IC) framework offers critical insights into multidimensional quality of life (QoL) determinants in older adults, systematic quantification of its clinical-social pathways remains limited. This cross-sectional study of 509 older adults (mean age 78.4±8.5 years) across community and institutional settings from China employed structural equation modeling with 5,000 bootstrap samples to examine pathways linking WHO-measured IC to EQ-5D-assessed QoL through frailty phenotype, Barthel Index (ADL), Visual Analog Scale (pain), Self-Rating Anxiety Scale, and Morse Fall Risk. The model demonstrated excellent fit (χ²/df=1.556, CFI=0.995, RMSEA=0.035). IC exhibited nonsignificant direct effects on QoL (β = 0.019, p = 0.416) versus robust indirect mediation through: Fall Risk (β = 0.090, 95%CI[0.065,0.120], 92.3% mediation), Frailty(β = 0.045[0.029, 0.060]), ADL(β = 0.030[0.012, 0.050]), Pain (β = 0.034[0.016, 0.050]), and Anxiety (β = 0.004[0.000,0.010]). Moderation analyses revealed social support enhanced IC’s effects via reduced fall risk (β = 0.015[0.005, 0.025]) and Anxiety, while caregiver availability strengthened Frailty (β = 0.027[0.012,0.042]) and ADL (β = 0.024[0.008, 0.040]) pathways. Comorbidity burden universally amplified pathway impacts (p < 0.001), most notably intensifying anxiety mediation by 90.0% (β = 0.900[0.843, 0.957]).These findings elucidate Intrinsic Capacity’s (IC) mechanisms influencing Quality of Life (QoL) through clinical mediators and social moderators. Social support and caregiver availability mitigate IC decline impacts, while comorbidity burden exacerbates effects, particularly amplifying anxiety mediation. Clinical priorities should emphasize fall risk screening, caregiver resource optimization, and comorbidity management. This study empirically validates the WHO IC framework, supporting precision health strategies that integrate biological resilience and social determinant optimization in aging populations.

